# Development and Characterization of Polymorphic EST-SSR and Genomic SSR Markers for Tibetan Annual Wild Barley

**DOI:** 10.1371/journal.pone.0094881

**Published:** 2014-04-15

**Authors:** Mian Zhang, Weihua Mao, Guoping Zhang, Feibo Wu

**Affiliations:** 1 Department of Agronomy, College of Agriculture and Biotechnology, Zijingang Campus, Zhejiang University, Hangzhou, P.R. China; 2 Center of Analysis and Measurement, Zhejiang University, Hangzhou, P.R. China; National Key Laboratory of Crop Genetic Improvement, China

## Abstract

Tibetan annual wild barley is rich in genetic variation. This study was aimed at the exploitation of new SSRs for the genetic diversity and phylogenetic analysis of wild barley by data mining. We developed 49 novel EST-SSRs and confirmed 20 genomic SSRs for 80 Tibetan annual wild barley and 16 cultivated barley accessions. A total of 213 alleles were generated from 69 loci with an average of 3.14 alleles per locus. The trimeric repeats were the most abundant motifs (40.82%) among the EST-SSRs, while the majority of the genomic SSRs were di-nuleotide repeats. The polymorphic information content (PIC) ranged from 0.08 to 0.75 with a mean of 0.46. Besides this, the expected heterozygosity (He) ranged from 0.0854 to 0.7842 with an average of 0.5279. Overall, the polymorphism of genomic SSRs was higher than that of EST-SSRs. Furthermore, the number of alleles and the PIC of wild barley were both higher than that of cultivated barley, being 3.12 *vs* 2.59 and 0.44 *vs* 0.37. Indicating more polymorphism existed in the Tibetan wild barley than in cultivated barley. The 96 accessions were divided into eight subpopulations based on 69 SSR markers, and the cultivated genotypes can be clearly separated from wild barleys. A total of 47 SSR-containing EST unigenes showed significant similarities to the known genes. These EST-SSR markers have potential for application in germplasm appraisal, genetic diversity and population structure analysis, facilitating marker-assisted breeding and crop improvement in barley.

## Introduction

Barley (*Hordeum vulgare* L.) is the fourth important cereal crop worldwide. With the rapid development of beer and feed industry, the demand for barley keeps increasing. However, during the long-term domestication of the cultivated barley, especially after the modern breeding and intensive cultivation, the genetic variation degraded significantly, resulting in missing lots of genes, including some rare alleles [1]. The monotonous genetic background of cultivated barley has become the bottleneck of the effectiveness of breeding, while the abundant diversity of wild barley can provide a pool of alleles for barley breeding and improvement [2], [3]. Morphological, archaeological cytogenetic and isozyme data revealed that wild barley on the Qinghai-Tibet Plateau is different from the Fertile Crescent wild barley [4]. Researches so far have shown even rich genetic diversity in Tibetan wild barley than in Ethiopian barley [5]. Novel germplasm has been identified from the Tibetan wild barley tolerant to drought, salinity and aluminum toxicity [6]–[8].

Increasing efficient molecular markers would be valuable in diversity analyses, resource conservation and beneficial alleles exploitation for wild barley. Comprehensive sets of expressed sequence tags (ESTs) sequences have been generated in many plants (http://www.ncbi.nlm.nih.gov/dbEST). The availability of increasing sequence databases enables the identification of functional genes with similar sequences in related species [9]. EST-based SSR markers (EST-SSRs) have been widely employed as powerful molecular genetic tools in a large number of cereal crop species due to their high level of transferability, close association to genes with known function, codominant inheritance, and low cost for development with available development from public databases [10]–[12]. Jaikishan et al. [13] used 25 EST-SSRs and 25 genomic SSRs to predict grain yield heterosis; multiple EST-SSRs were generated for wheat (*Triticum aestivum* L.) and these markers showed high transferability between wheat and the other crops, such as barley, maize, rice, and sorghum [14]–[16]. Up to date, polymorphic EST-SSRs were identified to establish *Hordeum chilense* evolutional relationships [17] and new EST-SSRs and genomic SSRs were complemented to the published Australian barley genetic maps [18]. However, to our knowledge, little work has been performed to develop EST-SSRs and apply them for population structure in Tibetan wild barley.

In the present study, with the objective of exploiting new SSRs from EST databases and confirming the published genomic SSRs in the Tibetan wild and cultivated barley accessions, 49 EST-SSRs and 20 genomic SSRs were developed and characterized. These markers can be utilized to evaluate the genetic variation and phylogenetic relationships of 96 barley genotypes. Furthermore, polymorphism, and genetic diversity in the Tibetan wild barley accessions were evaluated which would be particularly useful for identification of novel genes with traits of interest, and marker-assisted breeding in barley.

## Materials and Methods

### Plant materials

A total of 96 barley accessions were used in this study including 80 Tibetan annual wild barley from Qinghai-Tibet Plateau provided by Huazhong Agricultural University barley germplasm collection, and 16 cultivars from China which were stored at the Institute of Crop Science, Zhejiang University, Hangzhou, China (Table S1). These accessions were collected on public land. And no specific permits were required for the collection. Seeds were surface sterilized with 3% H_2_O_2_ for 30 minutes and thoroughly rinsed with distilled water, followed by germination in nutrient rich soil in an incubator (22/18°C, day/night) for 10 days. Total genomic DNA was extracted from barley leaves using the Plant Genomic DNA Kit (TianGen, Beijing, China).

### Sequence screening and primer designing

A total of 525999 barley ESTs were acquired from the EST database of GenBank (up to September 2012) (http://www.ncbi.nlm.nih.gov/Genbank/). Redundant sequences were removed from these ESTs using CD-HIT-EST (http://cd-hit.org) with the identity parameter of 95%. The presence of SSRs was screened using Simple Sequence Repeat Identification Tool (SSRIT) software (http://www.gramene.org/gramene/searches/ssrtool). The criteria for di-, tri-, tetra-, and penta-nucleotides were 10, 7, 5, and 4 repeat units, respectively. A total of 188 EST-SSRs were randomly selected and primers were designed using Primer5.0 with a length ranging from 18–22 bp, and product sizes of 100 to 300 bp. The reverse primers were marked with 6-FAM or HEX fluorescent dye at 5′ side for each pair. Based on the previous study of barley, 41 genomic SSR markers were selected and SSR primers were designed with the same criteria as mentioned above.

### PCR amplification and sequencing

PCR amplification was performed in a total of 20 µL reaction mixture that contained 1 µL of genomic DNA, 1 U ExTaq DNA polymerase (Takara Inc.), 2 µL of 10×Ex Taq Buffer (Mg^2+^ Plus), 0.2 mM dNTPs mix, 0.05 µM forward primers, 0.1 µM reverse primers and fluorescent primers (FAM or HEX).

The PCR protocol used was as follows: initial denaturation for 5 min at 94°C, followed by 5 cycles of denaturation for 30 s at 94°C, annealing for 30 s at 50°C, and extension for 30 s at 72°C, subsequently followed by 32 cycles of denaturation for 30 s at 94°C, annealing for 30 s at 55°C, extension for 30 s at 72°C, with a final extension for 10 min at 72°C and a 4°C holding temperature. PCR products were diluted and tested on a MegaBACE 1000 DNA analysis system (Amersham Biosciences, Piscataway, NJ) at the Center of Analysis and Measurement in Zhejiang University. The lengths of PCR fragments were calculated using the ET550-R size standard and Genetic Profiler version 2.2.

### Calculation of polymorphism

The polymorphism of EST- and genomic SSR alleles were scored for the presence (1) and absence (0) for 96 accessions. Alleles with frequency less than 5% (rare alleles) in the population were removed and considered as missing data for the polymorphism calculation and population structure analysis [19]. The genetic diversity was evaluated by the number of alleles (Na), the effective number of alleles (Ne), observed heterozygosity (Ho), and expected heterozygosity (He) using POPGENE v.1.31 [20]. Polymorphism information content (PIC) was calculated by applying software PIC_CALC version 0.6.

### Population structure

Population structure was assessed using the STRUCTURE software v2.3.3 based on the admixture model [21]. Models were tested for clusters (k) from 1 to 15, each with ten independent runs and 100,000 MCMC (Markov Chain Monte Carlo) iterations. The most likely number of clusters (k) was indicated by Δk, the change rate of the estimated log probability of the data (LnP[D]) [22].

### Gene function blast

EST-SSRs associated unigene sequences were blasted against the GenBank non-redundant (nr) protein database using BLASTX (http://www.ncbi.nlm.nih.gov/BLAST) with an expected value (E-value) of 10^−10^ for the function of polymorphic EST-SSRs.

## Results

### Characterization of polymorphic SSRs

In total, 69 SSR primer pairs, including 49 (26% out of 188) EST-SSRs and 20 (49% out of 41) genomic SSRs (Tables 1 and 2), showed polymorphism among 96 accessions. A total of 213 alleles were generated from 69 loci with an average of 3.14 alleles per locus. The ratio of the EST-SSR repeat motifs was not equally distributed. The di-, tri-, tetra-, and penta-nucleotides accounted for 16.32%, 40.82%, 26.53%, and 16.32%, respectively. Whilst most of the genomic SSRs selected were composed of dinucleotide repeats. According to the results of POPGENE for the 69 SSRs, the observed number of alleles per locus (Na) ranged from 2 to 6 (mean = 3.14) and the effective number of alleles per locus (Ne) varied from 1.09 to 4.54 (mean = 2.30). The average Na was 3.12 and 2.59 for wild and cultivated barley, respectively (Table 3). Besides this, the polymorphic information content (PIC) ranged from 0.08 to 0.75 with a mean of 0.46, and the PIC of wild barley was higher than that of cultivars with 0.44 *vs* 0.37. The expected heterozygosity (He) ranged from 0.0854 to 0.7842 with an average of 0.5279, while the observed heterozygosity (Ho) ranged from 0 to 0.766 with an average of 0.1677. As an indicator of genetic diversity, the average He was 0.5098 in wild barley accessions and 0.4333 in cultivated accessions.

**Table 1:** Characterization of 49 polymorphic EST-SSR makers in barley (*Hordeum vulgare*L.).

Primer	SSR motif	Primer sequence (5′-3′)	Expected size(bp)	Na	Ne	Ho	He	PIC
		F/R						
P181	(GAGAG)4	GTCGTCTCCCTCCCTTCA	227	5	3.23	0.1979	0.6944	0.6379
		CATTGCCAGCACTGTTTC						
P129	(GCC)7	CGAGGAGTTCGAGGTGGA	260	4	3.26	0.6042	0.697	0.635
		ACTCTGCGTCCCAGTTCTT						
P184	(TGC)9	CCTACCAAACAACGGAATA	276	4	3.04	0.1053	0.6746	0.6232
		CAGCCAGAAGGTCTACGA						
P50	(AATC)5	ACAAGCAGATCACCGACG	215	3	2.95	0.2727	0.6649	0.5868
		AACCCGACTGAACAAATAAT						
P91	(TC)13	CGAGGCTCCTCATCTCCT	211	3	2.89	0.3542	0.657	0.5796
		CCAGCATCGTCGCAAACT						
P8	(AG)15	TCGTTGATCCGAACTTTACC	197	3	2.82	0.2188	0.6484	0.5735
		CACCGCAGACGCTGAGTA						
P29	(ATAC)13	CTGCTTAGTTCTAGGAGGCT	140	3	2.57	0.1935	0.6141	0.5391
		CTCGGTTCGATTGTTCAT						
P103	(CTG)9	CATTTGGCATTGGTTGAT	100	3	2.53	0.0104	0.6073	0.5362
		AGTTCTTCTTCGCTGGAA						
P32	(GATG)6	GCAGAATGGCAGAAACAG	233	3	2.6	0.0957	0.6187	0.5352
		CAAGAATGAGCGAAAGGT						
P168	(TTC)7	TTCCTCCAGTCCTTCTCC	169	4	2.49	0.3511	0.6013	0.5344
		CTGCTGCTACCGTTCTTAT						
P99	(ATC)7	GATGTGATCTGATGCCATTT	273	3	2.46	0.25	0.5966	0.5266
		TTTCTTCGGTGTTCTTTCC						
P152	(CT)11	ACCAAGCCCACGAGTAGCA	251	3	2.4	0.0521	0.5867	0.5186
		CGACCCGAGGACGACAGAT						
P144	(CT)11	CTTCGTTCCCTCCTCACC	134	5	2.19	0.4545	0.5473	0.5127
		TCCGCTTCCACGATTGAC						
P121	(TACAT)4	CCCAGGAATAAGAACAGACAC	287	4	2.25	0.3684	0.5587	0.4971
		CACCGCCTAATAGCAACAA						
P34	(CTTC)6	GGCGAGGAACTGTTGTTG	252	3	2.33	0.2083	0.5738	0.4898
		GATCGGCTTCATCGTCTACT						
P101	(ATC)12	CCCCGTATAAACCACCCA	245	3	2.18	0.2556	0.5439	0.4827
		GGCAGAACTTCAGCACCC						
P149	(AGC)9	CTTGGCACGCTTTGTTTG	259	3	2.17	0.191	0.5431	0.478
		ACTTTCCCACGGCATCAG						
P150	(GAGC)5	TAAGTAGGTTTGAGGAAGGGAA	265	3	2.22	0.1149	0.5533	0.4693
		CAACATAGACAAGGTGCTGGA						
P83	(AAGAA)4	CTCGGCAAACAGAGGACA	278	4	2.21	0.2083	0.5506	0.468
		TTGTAGCAGCGGATGGTC						
P30	(ATGT)12	ACTGCCACTCCATTTAGG	241	3	2.16	0.1684	0.5407	0.4626
		CTGTCGTAGGCTTGCTTT						
P63	(AGC)9	GGCTTGGCACGCTTTGTT	259	3	2.07	0.1146	0.5206	0.4573
		TTTCCCACGGCATCAGTC						
P90	(GAT)7	CGCAAGCCACAGAGCACA	177	3	2.12	0.1146	0.5301	0.4507
		TCCGTCCGTTCGTCCATC						
P9	(AC)11	ATCACAAACAGCCACTGTCCTA	111	4	2.01	0.2812	0.5047	0.4388
		GTGGTGAACCTTGCCCTTG						
P3	(GA)10	GCGAGGATGATGTATAAACCG	132	4	1.95	0.3077	0.4887	0.4256
		TGCATTCTGTGCCCTAACTAA						
P45	(GGTT)5	CCCACAACACCAACAAAC	229	3	2.08	0.1771	0.5219	0.413
		GCCCGTAGAATGAACAAGTA						
P55	(CTG)9	TTGATGGAGAAGGAGCAT	264	3	1.78	0.0319	0.4419	0.3926
		ACATAGTAGGATAGATAGACCC						
P105	(CCTCG)4	GCGACTACCAGGACGACAA	297	3	1.78	0.0632	0.4415	0.381
		CACCGACCGATACAGACAGA						
P56	(CTG)7	AGTGATCTGAGGCGGTAT	176	2	1.99	0.1875	0.5007	0.374
		CGTACGTCCAATGTTGTC						
P66	(CTCTT)4	CAAATGTGCCAGTAGAAA	293	2	1.99	0.234	0.5006	0.374
		GGATGAGTTGCAGGTGAT						
P67	(TTG)12	AGAAACAAACAGACAGACCCAT	284	2	1.97	0.5729	0.496	0.3717
		ATTCCACCACCGTCACCA						
P180	(CAG)8	ATTCTCGCCGCCAACAACT	217	2	1.97	0.2	0.4946	0.371
		CCACGTAGAAAGGGAGGGTCA						
P80	(GGTTG)4	ACTCCTGCTGCTGCTGAC	149	2	1.95	0.3229	0.4903	0.3688
		CGGTATTAGGCGACTCTTC						
P57	(AATA)5	ATAACAGCCGTTGATGAG	260	2	1.94	0.2604	0.4869	0.367
		GATCCGTTCCACAAACAT						
P54	(ATC)7	CAGCACCACTACTAATCAAGAA	245	2	1.93	0	0.4849	0.366
		GCCACCAACAAGACCTCC						
P137	(GAAGA)4	AGAGGACAAGCCAAGGAAG	161	2	1.91	0.1739	0.479	0.3629
		CACGGAAACGGAACAAAA						
P106	(CTG)8	CGAGCCGTTGCTTAGGTC	206	2	1.85	0.1383	0.4612	0.3535
		TCTACTGCCAGGGCGTGA						
P139	(GCAT)5	ACTCACATAGTAATCGAAGGG	287	2	1.83	0.4896	0.4568	0.3512
		GGGCAAGAACGAATCTCC						
P186	(CTGA)5	GGTAGTTCCGCCATCAGA	177	2	1.72	0.2604	0.4197	0.3303
		CCTCCTGTGGACGAAGAT						
P187	(GCACA)4	CTCGGACGACCATTTATT	209	2	1.7	0.1875	0.4154	0.3278
		TTCAAAGTTCAAGGGTGC						
P53	(CCAA)5	AGGGAAAGAAATCCTAAC	224	2	1.63	0.0968	0.3902	0.3128
		TTGACTTGCTTATACACCT						
P13	(AT)19	CACATGCGTTAGTGTCCC	298	2	1.63	0	0.3899	0.3126
		GCGATTATCTTCGTCCAG						
P16	(TG)11	CGAGCAGGCATAGCCATAT	256	3	1.44	0.069	0.3097	0.2853
		GACGCTGAGTACGTTGAGGT						
P61	(GCA)8	CAAATGGAGCCAAGCAAC	235	2	1.47	0.1828	0.3204	0.2679
		CCATCCTTGACGCACATC						
P81	(CTG)8	GCAGGATAGGCGACACTC	141	2	1.38	0.1333	0.2793	0.2392
		GAGACGGAGAAGGAGCAG						
P185	(CGG)8	AAACGGCTTTCACATCTCCC	201	2	1.38	0.0625	0.2792	0.2392
		CGCCCAAACAAGTCCTCC						
P120	(AGC)7	GAAATACTCCCAGGACAGC	249	2	1.33	0.0106	0.2473	0.2157
		AGCAAGTGCCAGTTCTACC						
P100	(CACG)6	CACATAAACAACCGAACCAA	245	2	1.23	0.0208	0.1876	0.1693
		CGACATACGCAGGGAGTG						
P21	(GAC)7	AACCTATGCCGCCTACTT	241	2	1.11	0.0417	0.0993	0.0939
		CCACCCGTCCACTCTTTT						
P44	(GCAA)5	AGTCCCGTAAACCTACCTGAG	165	2	1.09	0	0.0854	0.0813
		TGCCGGAGAATGTAATCG						

Note: Na, number of alleles; Ne, number of effective alleles; Ho, observed heterozygosity; He, expected heterozygosity; PIC, polymorphic information content.

**Table 2:** Characterization of 20 genomic-SSR makers in barley.

Primer	SSR motif	Primer sequence (5′-3′)	Expected size (bp)	Na	Ne	Ho	He	PIC
		F/R						
S40	(AT)29	ACACCTTCCCAGGACAATCC	182	6	4.54	0.022	0.7842	0.748
		CAGAGCACCGAAAAAGTCTGTA						
S22	(GT)13,(AG)19	AAGCTCTTTCTTGTATTCGTG	158	5	4.09	0.0526	0.7595	0.7162
		GTCCATACTCTTTAACATCCG						
S18	(CT)28	CTGGGATTGGATCACTCTAA	107	5	3.9	0.0211	0.7474	0.7016
		AAAACAAGTACTGAAAATAGGAGA						
S7	(AC)20	ATAGATCACCAAGTGAACCAC	177	5	3.49	0.0833	0.7175	0.6776
		GGTTATCACTGAGGCAAATAC						
S37	(CT)18	CCGACAACATGCTATGAAGC	131	5	3.35	0.0521	0.7049	0.6596
		CTGCAGCAAATACCCATGTG						
S2	(AC)7T(CA)15 (AT)9	CCATCAAAGTCCGGCTAG	215	4	3.32	0.0326	0.703	0.6504
		GTCGGGCCTCATACTGAC						
S11	(AG)15	TCCATGATGATGTGTGCATAGA	173	5	3.01	0.0909	0.672	0.6121
		CGGATCCCAACAAACACAC						
S4	(AT)6(AC)16	GCTATGGCGTACTATGTATGGTTG	173	4	3.04	0.0549	0.6749	0.6106
		TCACGATGAGGTATGATCAAAGA						
S41	(TG)8	AGTATGGGGAATTTATTTGG	136	4	2.79	0.0312	0.6455	0.5864
		GCTGCAAAGTATGACAATATG						
S25	(CT)24	TTTGTGACATCTCAAGAACAC	158	4	2.77	0.1889	0.6428	0.5845
		TGACAAACAAATAATCACAGG						
S38	(GA)17(GA)7	CTATCACACGACGCAACATG	169	5	2.73	0.5106	0.6376	0.5828
		CCTGAGAAAGAAAGCGCAAC						
S30	(GC)5GGG (GT)16	CAAATCAATCAAGAGGCC	153	3	2.74	0	0.6384	0.5615
		TTTGAAGTGAGACATTTCCA						
S21	(AG)7C(AG)30-(AG)6	GGGAACTTGCTAATGAAGAG	150	3	2.67	0	0.6284	0.5546
		AATGTAAGGGAGTGTCCATAG						
S19	(AG)19	CCCTAGCCTTCCTTGAAG	135	3	2.46	0.0316	0.5973	0.5292
		TTACTCAGCAATGGCACTAG						
S29	(GT)16	AGAATCAAGATCGACCAAAC	124	4	2.19	0.0233	0.5464	0.5027
		AAAAACATGAACCGATGAA						
S15	(CT)16	ATTCATCGATCTTGTATTAGTCC	174	3	2.16	0.0319	0.5391	0.4749
		ACATCATGTCGATCAAAGC						
S31	(CT)21	CTATTTTCTAATGCTTGGACC	149	3	2.18	0.0947	0.5437	0.4647
		TGTCTAGTTCATCATCATTGC						
S36	(CA)9	GGATTTTCTCAAGAACACTT	239	3	2.13	0.766	0.5324	0.4597
		GCGTGAGTGCATAACATT						
S1	(AC)11	GTCCTTTACGCATGAACCGT	138	3	2.1	0.0316	0.5256	0.4547
		ACATACGCCAGACTCGTGTG						
S8	(AC)13(AT)9	GCTCTCTCTCAGAAAAATGAA	177	3	1.63	0.0444	0.3899	0.3492
		GAATTATTCTAGGGCTGTGAA						

**Table 3:** Polymorphism of SSR makers in Tibetan wild and cultivated barley.

	No. of alleles		PIC		He			No. of alleles		PIC			He
Marker	Wild	Cultivated	Wild	Cultivated	Wild	Cultivated	Marker	Wild	Cultivated	Wild	Cultivated	Wild	Cultivated
P3	4	2	0.450	0.156	0.5259	0.1754	P129	4	4	0.581	0.658	0.6544	0.7359
P8	3	3	0.582	0.482	0.6597	0.5565	P137	2	1	0.374	0	0.5019	0
P9	4	2	0.467	0.110	0.5454	0.1210	P139	2	2	0.332	0.366	0.4226	0.4980
P13	2	2	0.271	0.371	0.3251	0.5081	P144	5	2	0.523	0.346	0.5553	0.4598
P16	3	3	0.256	0.375	0.2757	0.4456	P149	3	2	0.495	0.332	0.5609	0.4345
P21	2	1	0.110	0	0.1179	0	P150	3	3	0.460	0.456	0.5357	0.5701
P29	3	3	0.484	0.520	0.5637	0.6048	P152	3	3	0.442	0.450	0.5048	0.5222
P30	3	3	0.431	0.398	0.4908	0.4758	P168	4	2	0.571	0.258	0.6388	0.3145
P32	3	2	0.542	0.315	0.6201	0.4046	P180	2	1	0.374	0	0.5019	0
P34	3	2	0.507	0.366	0.5878	0.4980	P181	4	3	0.618	0.478	0.6731	0.5544
P44	2	2	0.075	0.110	0.0783	0.1210	P184	4	3	0.567	0.468	0.6177	0.5484
P45	3	3	0.365	0.294	0.4623	0.3306	P185	2	2	0.229	0.283	0.2653	0.3528
P50	3	2	0.545	0.305	0.6285	0.3871	P186	2	2	0.345	0.195	0.4458	0.2258
P53	2	2	0.288	0.374	0.3503	0.5149	P187	2	2	0.347	0.110	0.4500	0.1210
P54	2	2	0.372	0.258	0.4981	0.3145	S1	3	2	0.488	0.195	0.5651	0.2258
P55	3	2	0.382	0.359	0.4237	0.4839	S2	4	3	0.655	0.440	0.7106	0.5425
P56	2	2	0.371	0.359	0.4953	0.4839	S4	4	3	0.615	0.561	0.6740	0.6587
P57	2	2	0.347	0.283	0.4500	0.3528	S7	4	2	0.583	0.359	0.6318	0.4839
P61	2	2	0.280	0.195	0.3389	0.2258	S8	3	3	0.275	0.528	0.2988	0.6323
P63	3	2	0.475	0.323	0.5386	0.4173	S11	5	3	0.640	0.327	0.6996	0.3730
P66	2	2	0.364	0.258	0.4821	0.3145	S15	3	3	0.428	0.563	0.4927	0.6621
P67	2	2	0.375	0.305	0.5024	0.3871	S18	5	4	0.704	0.592	0.7511	0.6694
P80	2	2	0.372	0.323	0.4981	0.4173	S19	3	3	0.457	0.412	0.5197	0.4966
P81	2	2	0.248	0.195	0.2916	0.2258	S21	3	3	0.521	0.460	0.5902	0.5652
P83	4	3	0.473	0.438	0.5550	0.5423	S22	5	5	0.683	0.687	0.7344	0.7581
P90	3	3	0.378	0.544	0.4479	0.6371	S25	4	4	0.560	0.515	0.6307	0.6000
P91	3	3	0.587	0.327	0.6650	0.3730	S29	4	4	0.429	0.607	0.4713	0.7059
P99	3	2	0.549	0.305	0.6223	0.3871	S30	3	3	0.528	0.354	0.5985	0.4113
P100	2	2	0.110	0.337	0.1179	0.4435	S31	3	2	0.499	0.110	0.5846	0.1210
P101	3	2	0.499	0.315	0.5637	0.4046	S36	3	3	0.423	0.548	0.5021	0.6414
P103	3	2	0.470	0.371	0.5252	0.5081	S37	5	3	0.584	0.555	0.6318	0.6452
P105	3	2	0.380	0.195	0.4634	0.2258	S38	5	4	0.510	0.626	0.5732	0.7011
P106	2	2	0.329	0.349	0.4185	0.4657	S40	5	3	0.694	0.363	0.7422	0.4203
P120	2	2	0.159	0.359	0.1749	0.4839	S41	4	4	0.530	0.483	0.5809	0.5565
P121	4	3	0.485	0.367	0.5584	0.4529							
							**Average**	**3.12**	**2.59**	**0.441**	**0.373**	**0.5098**	**0.4333**

### Gene functions of the 49 unigene sequences containing polymorphic EST-SSRs

Functions of the 49 polymorphic EST-SSRs were determined and 47 unigenes showed significant similarities to the known genes (Table 4), for instance, zinc finger protein MAGPIE, transcription factor LAF1, photosystem II reaction center PSB28 protein, xyloglucan endotransglycosylase (XET), and protein kinase APK1B. In addition, the results revealed that the most annotated proteins were from *Triticum urartu* (17, 36.2%), and the species *Hordeum vulgare* and *Aegilops tauschii* accounted for the same percentage (11, 23.4%).

**Table 4:** The putative proteins identified by BLASTX of 49 unigene sequences containing polymorphic EST-SSRs.

Primer	Accession No.	Putative protein	Organism	E-value
P181	CA032876.1	Hypothetical protein TRIUR3_30088	*Triticum urartu*	4.00E-51
P129	CV063130.1	Putative SKP1 protein	*T.aestivum*	1.00E-77
P184	CB858539.1	Hypothetical protein TRIUR3_19075	*T.urartu*	1.00E-46
P50	DN178534.1	UCW116, putative lipase	*H. vulgare* subsp. *vulgare*	3.00E-125
P91	FD524685.1	Putative syntaxin-131	*Aegilops tauschii*	1.00E-93
P8	AL506646.1	Zinc finger protein MAGPIE	*T.urartu*	4.00E-41
P29	AV943994.1	RNA polymerase sigma factor rpoD	*T.urartu*	7.00E-116
P103	CA009356.1	GID1-like gibberellin receptor	*H. vulgare* subsp. *vulgare*	4.00E-04
P32	EX593207.1	Disease resistance protein RGA2	*Aegilops tauschii*	8.70E-02
P168	BU997138.1	Hypothetical protein TRIUR3_09517	*T.urartu*	1.00E-04
P99	GH218162.1	Two-component response regulator ARR9	*T.urartu*	2.00E-64
P152	AV938130.1	Predicted protein	*H. vulgare* subsp. *vulgare*	1.10E-01
P144	EX598444.1	No hit	*-*	-
P121	CK569829.1	ACC oxidase	*H. vulgare*	9.00E-74
P34	DN186304.1	Predicted: UDP-glucose 6-dehydrogenase-like	*Brachypodium distachyon*	5.00E-65
P101	GH223749.1	FT-like protein	*H. vulgare* subsp. *vulgare*	1.00E-45
P149	EX583185.1	Condensin-2 complex subunit G2	*T.urartu*	5.00E-54
P150	FD519288.1	Curcuminoid synthase	*T.urartu*	5.00E-59
P83	FD527549.1	Putative pectinesterase 53	*Aegilops tauschii*	1.00E-76
P30	DN177250.1	Hypothetical protein F775_31773	*Aegilops tauschii*	1.00E-05
P63	EX577085.1	Condensin-2 complex subunit G2	*T.urartu*	6.00E-69
P90	FD528427.1	Photosystem II reaction center PSB28 protein	*T.urartu*	2.00E-83
P9	AL505258.1	Hypothetical protein f775_27232	*Aegilops tauschii*	6.00E-113
P3	BJ547928.1	Hypothetical protein TRIUR3_27885	*T.urartu*	1.00E-113
P45	FD523777.1	Hypothetical protein OsI_14737	*Oryza sativa* Indica Group	3.00E-50
P55	AL505545.1	No hit	*-*	-
P105	CA014373.1	Eukaryotic translation initiation factor 1A	*Leymus chinensis*	5.00E-72
P56	EX584572.1	Hypothetical protein F775_08651	*Aegilops tauschii*	2.00E-37
P66	FD518055.1	Predicted: protein LOC100843116	*B.distachyon*	5.00E-51
P67	FD520223.1	Hypothetical protein TRIUR3_27901	*T.urartu*	8.00E-36
P180	CA030489.1	Hypothetical protein TRIUR3_23016	*T.urartu*	4.00E-73
P80	FD523499.1	Casein kinase I-2-like protein	*A.tauschii*	1.00E-75
P57	EX599270.1	Hypothetical protein ZEAMMB73_419738	*Zea mays*	7.00E-56
P54	AL500476.1	PM2	*H. vulgare* subsp. *vulgare*	5.00E-67
P137	DN180922.1	PREDICTED: protein LOC100846358	*B.distachyon*	2.00E-02
P106	CA031374.1	OSJNBa0074L08.11	*Oryza sativa* Japonica Group	1.00E-46
P139	AL501810.1	GDSL esterase/lipase	*A.tauschii*	3.00E-40
P186	CB864664.1	Protein kinase APK1B, chloroplastic	*A.tauschii*	4.00E-50
P187	CB864737.1	Inactive ubiquitin carboxyl-terminal hydrolase 54	*T.urartu*	1.00E-17
P53	EH090859.1	TBC1 domain family member 15	*A.tauschii*	2.00E-57
P13	CK569261.1	Hypothetical protein TRIUR3_25268	*T.urartu*	3.50E-01
P16	CB873886.1	Phospholipid transfer protein precursor	*H. vulgare* subsp. *vulgare*	2.00E-43
P61	EX573461.1	Predicted protein	*H. vulgare* subsp. *vulgare*	6.00E-60
P81	FD521065.1	Predicted protein	*H. vulgare* subsp. *vulgare*	1.00E-81
P185	CB860073.1	Peptide transporter PTR2	*A.tauschii*	5.00E-60
P120	CK569159.1	Xyloglucan endotransglycosylase (XET)	*H. vulgare* subsp. *vulgare*	5.00E-69
P100	GH216950.1	Rho GDP-dissociation inhibitor 1	*T.urartu*	7.00E-69
P21	CK122115.1	Predicted protein	*H. vulgare* subsp. *vulgare*	5.00E-116
P44	CV063055.1	Transcription factor LAF1	*T.urartu*	3.00E-70

### Population structure and genetic distance

**Figure 1:**
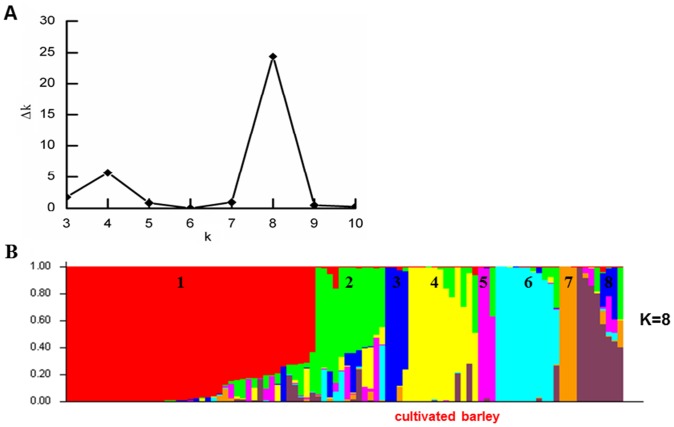
Δk and population structure. Estimation of the likelihood of clusters (k) for the most appropriate subpopulations (Δk) (A), and the population structure of 96 barley accessions in k = 8 clusters (B).

To detect the population structure in the 96 barley genotypes, we performed STRUCTURE program for Bayesian clustering analysis using 69 SSR markers, assuming that the number of populations (K) ranged from 1 to 15. The highest log likelihood score (Δk) was at K = 8 (Figure 1A), indicating that the most suitable number of subpopulations was eight. The frequency of each accession assigned to a subpopulation was shown in Table S1. If the threshold of frequency was set at 0.5, only six accessions were defined as admixed. However, about 80% of the accessions can be derived from the subpopulations when the threshold was at 0.7. The output of structure analysis demonstrated that wild and cultivated barleys were assigned to different subpopulations (Figure 1B). Most of the cultivated barleys were classified into the subpopulation 4, except for A74, Tadmor, B1342 and B1031. Fifty percent of the wild barley accessions studied were assigned to subpopulation 1.

**Figure 2:**
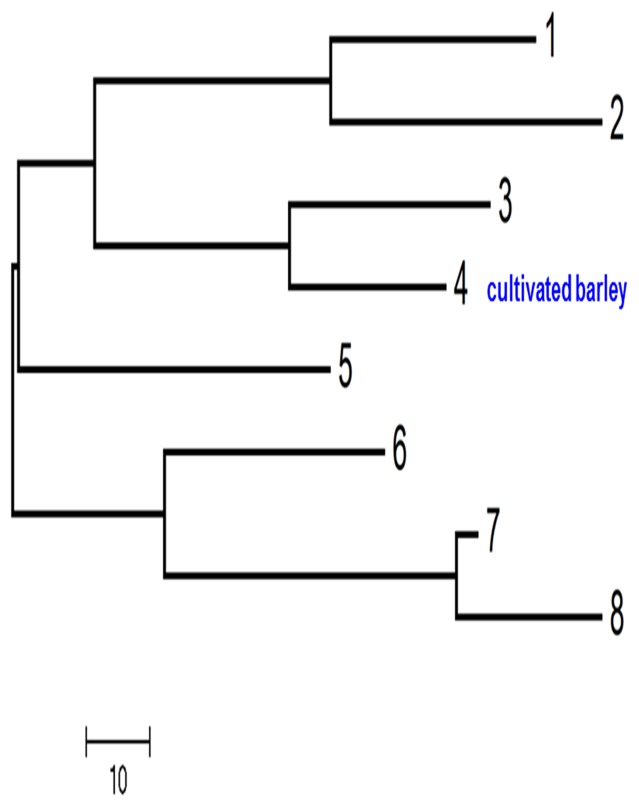
The dendrogram of the eight subpopulations according to the genetic distance using UPGMA clustering analysis.

According to the values of genetic distance of the eight subpopulations, we get the dendrogram showing the genetic relationship of the subpopulations via UPGMA clustering analysis (Figure 2). The dendrogram showed that the subpopulation 3 was most close to the cultivated barleys (subpopulation 4) with the genetic distance of 132.188. The subpopulation 7 had the largest genetic distance (165.167) with the cultivated subpopulation.

## Discussion

In recent years, different kinds of molecular markers have been used widely, including marker-assisted breeding, study of genetic relationships between populations, and screening candidate genes associated with the target traits [23]. The simple sequence repeats (SSRs) are increasingly important due to their high polymorphism and convenient techniques. However, EST-SSRs are superior to genomic SSRs for their transcriptional sequence and suitable application in cross-species [24]. In the present study, we developed 49 EST-SSR and 20 genomic SSR markers for wild barley. These novel EST-derived markers will be a valuable resource for tagging and mapping of genes related to agronomic and stress-resistant traits of interest. In addition, these markers are advantageous for identifying functional diversity of unique adaptive germplasm because of their genic function.

In many plants, the di- and tri-nucleotides repeat motifs were the major types, but the predominant motifs were different in various species [25], [26]. In our research, the tri-meric repeats were the most abundant motifs (40.82%), followed by the tetra-meric repeats accounted for 26.53%, and the di-meric and penta-meric repeat motifs were at the same frequency (16.32%).The polymorphism of SSRs can be divided into three degrees: high (PIC>0.5), medium (0.5>PIC>0.25) or low (PIC<0.25) [27]. In our study, the genetic diversity of genomic SSRs was higher than the EST-SSRs, with the mean PIC value of 0.57 (high) and 0.41 (medium), respectively, resulting in the general medium polymorphism (mean = 0.46). This finding was in line with previous results, and the lower level of polymorphism of EST-SSRs might be due to the selection against the variation in the conserved regions of the EST-SSRs [28]. Moreover, the expected levels of heterozygosity at EST-SSRs were also not as high as that of genomic SSRs, ranging from 0.0854 to 0.697 *vs* 0.3899 to 0.7842. Pompanon et al. [29] contributed the deficiency of heterozygosity to the primer problems, the deletion of alleles and appearance of invalid alleles at the annealing points.

Studies of the genetic variation in barley suggested that Tibetan wild barley showed higher polymorphism than cultivated barley [30]–[32]. The results of our study were consistent with the previous studies. The number of alleles and the PIC of wild barley were both higher than that of cultivated barley, being 3.12 *vs* 2.59 and 0.44 *vs* 0.37. The expected heterozygosity (He) showed the same trend, with 0.5098 and 0.4333 for wild and cultivated barley, respectively. The richness of genetic diversity in Tibetan wild barley may be the source of novel genes contributing to the tolerance of biotic and abiotic stresses, which is important in the barley breeding.

BLASTX analysis indicated that 47 (96%) of the 49 unigenes containing EST-SSRs can be matched to at least one important proteins in the NCBI nr protein database. For futher study, we can search the candidate genes of interest via association analysis referring to the function of markers in the metabolism pathways. Furthermore, these EST-SSR markers can be utilized as affirmative markers for comparative studies in the related species, for example, *Triticum urartu* and *Aegilops tauschii*.

In the present investigation, the findings of population structure analysis demonstrated that the developed EST-SSRs and genomic SSRs could distinguish between the cultivated and wild barley genotypes clearly. The 96 genotypes were divided to eight subpopulations. The subpopulation 3 (XZ161, XZ163, XZ165, XZ168) was most closely related to the cultivated barley (subpopulation 4), and the subpopulation 7 (XZ120, XZ151, XZ153) and the cultivated barleys were two most genetically distant populations. The genetic relation of the subpopulations suggested that the subpopulation 3 contained the most domesticated genotypes among the studied wild barley. Futhermore, the other subpopulations of wild barley, especially subpopulation 7, may be the important germplasm resource for the improvement of cultivars tolerant of abiotic and biotic stresses. These results were consistent with recent clustering studies in the Tibetan wild barley genotype using DArT markers and SNPs[3]. This indicates that the cluster analysis using EST-SSR and SSR markers is an effective way to determine the structure of populations and can constitute a solid foundation for the genetic variation study.

## Conclusion

The 49 novel EST-SSRs and 20 genomic SSR markers developed from 96 barley genotypes were highly polymorphic and could be employed to examine genetic diversity, evolution, linkage mapping, comparative genomics, and population structure. The Tibetan wild barley showed higher genetic variation than cultivated barley, and the cultivated subpopulation could be separated from the wild barley clearly. For further studies, these developed markers could be useful in identifying trait-marker association of interest in the marker-assisted breeding programs in barley.

## Supporting Information

Table S1 **List of 96 genotypes used in this study and their inferred subpopulations with k = 8.**
(DOCX)
