# Supplementary material for: Development and Characterization of Polymorphic EST-SSR and Genomic SSR Markers for Tibetan Annual Wild Barley
Source: PLoS One. 2014 Apr 15;9(4):e94881. doi: 10.1371/journal.pone.0094881 (PMC3988095; doi:10.1371/journal.pone.0094881)
Supplement: Table S1 — List of 96 genotypes used in this study and their inferred subpopulations with k = 8. (DOCX) [file pone.0094881.s001.docx]

**Table S1**. List of 96 genotypes used in this study and their inferred subpopulations with k=8.

| Genotype | wild/cultivated | Origin | Subpopulation | Frequence | Genotype | wild/cultivated | Origin | Subpopulation | Frequence |
| --- | --- | --- | --- | --- | --- | --- | --- | --- | --- |
| XZ3 | wild | *H.a* | 2 | 0.696 | XZ130 | wild | *H.s* | 1 | 0.806 |
| XZ5 | wild | *H.a* | admixed | 0.443 | XZ131 | wild | *H.s* | 1 | 0.994 |
| XZ8 | wild | *H.s* | admixed | 0.401 | XZ133 | wild | *H.a* | 6 | 0.997 |
| XZ9 | wild | *H.s* | 8 | 0.573 | XZ134 | wild | *H.s* | 6 | 0.998 |
| XZ12 | wild | *H.s* | 2 | 0.639 | XZ138 | wild | *H.a* | 6 | 0.994 |
| XZ16 | wild | *H.a* | 2 | 0.611 | XZ139 | wild | *H.s* | 1 | 0.993 |
| XZ19 | wild | *H.s* | admixed | 0.444 | XZ140 | wild | *H.a* | 6 | 0.998 |
| XZ23 | wild | *H.s* | admixed | 0.486 | XZ141 | wild | *H.a* | 6 | 0.998 |
| XZ25 | wild | *H.a* | 8 | 0.919 | XZ142 | wild | *H.s* | 1 | 0.997 |
| XZ26 | wild | *H.s* | 2 | 0.942 | XZ143 | wild | *H.s* | 1 | 0.961 |
| XZ34 | wild | *H.s* | 2 | 0.72 | XZ144 | wild | *H.a* | 5 | 0.996 |
| XZ45 | wild | *H.a* | 6 | 0.899 | XZ145 | wild | *H.s* | 1 | 0.992 |
| XZ51 | wild | *H.s* | 1 | 0.712 | XZ147 | wild | *H.s* | 1 | 0.97 |
| XZ52 | wild | *H.s* | admixed | 0.494 | XZ148 | wild | *H.s* | 1 | 0.877 |
| XZ54 | wild | *H.s* | 1 | 0.722 | XZ150 | wild | *H.s* | 1 | 0.997 |
| XZ57 | wild | *H.s* | 1 | 0.841 | XZ151 | wild | *H.s* | 7 | 0.998 |
| XZ58 | wild | *H.s* | 1 | 0.722 | XZ153 | wild | *H.s* | 7 | 0.998 |
| XZ60 | wild | *H.s* | 1 | 0.825 | XZ154 | wild | *H.s* | 1 | 0.989 |
| XZ62 | wild | *H.s* | 5 | 0.631 | XZ156 | wild | *H.s* | 1 | 0.997 |
| XZ63 | wild | *H.s* | 6 | 0.876 | XZ158 | wild | *H.s* | 1 | 0.98 |
| XZ66 | wild | *H.a* | 2 | 0.665 | XZ161 | wild | *H.s* | 3 | 0.995 |
| XZ69 | wild | *H.s* | 1 | 0.831 | XZ163 | wild | *H.s* | 3 | 0.861 |
| XZ77 | wild | *H.s* | 1 | 0.79 | XZ165 | wild | *H.s* | 3 | 0.997 |
| XZ82 | wild | *H.s* | 1 | 0.633 | XZ166 | wild | *H.s* | 1 | 0.74 |
| XZ86 | wild | *H.s* | 6 | 0.995 | XZ168 | wild | *H.s* | 3 | 0.725 |
| XZ88 | wild | *H.s* | 1 | 0.827 | XZ174 | wild | *H.s* | 2 | 0.638 |
| XZ89 | wild | *H.s* | 1 | 0.996 | XZ176 | wild | *H.a* | 8 | 0.802 |
| XZ95 | wild | *H.s* | 1 | 0.997 | XZ179 | wild | *H.a* | 6 | 0.957 |
| XZ97 | wild | *H.s* | 1 | 0.996 | XZ180 | wild | *int* | admixed | 0.41 |
| XZ98 | wild | *H.s* | 1 | 0.97 | XZ183 | wild | *int* | 8 | 0.858 |
| XZ99 | wild | *H.s* | 1 | 0.972 | XZ186 | wild | *H.s* | 1 | 0.72 |
| XZ100 | wild | *int* | 5 | 0.963 | XZ101 | wild | *H.s* | 1 | 0.824 |
| XZ102 | wild | *H.s* | 1 | 0.997 | ZJU3 | cultivated | *H.v* | 4 | 0.973 |
| XZ104 | wild | *H.s* | 1 | 0.996 | A74 | cultivated | *H.v* | 2 | 0.591 |
| XZ108 | wild | *H.s* | 1 | 0.806 | Tadmor | cultivated | *H.v* | 2 | 0.732 |
| XZ109 | wild | *H.s* | 1 | 0.942 | B1034 | cultivated | *H.v* | 4 | 0.993 |
| XZ111 | wild | *H.s* | 1 | 0.997 | B1031 | cultivated | *H.v* | 8 | 0.994 |
| XZ112 | wild | *H.s* | 1 | 0.847 | B1016 | cultivated | *H.v* | 4 | 0.699 |
| XZ113 | wild | *H.s* | 1 | 0.996 | B1064 | cultivated | *H.v* | 4 | 0.775 |
| XZ115 | wild | *H.s* | 1 | 0.991 | B1157 | cultivated | *H.v* | 4 | 0.509 |
| XZ116 | wild | *H.s* | 1 | 0.996 | B1100 | cultivated | *H.v* | 4 | 0.669 |
| XZ117 | wild | *H.s* | 1 | 0.997 | B1130 | cultivated | *H.v* | 4 | 0.774 |
| XZ118 | wild | *H.s* | 1 | 0.996 | Hua30 | cultivated | *H.v* | 4 | 0.996 |
| XZ120 | wild | *H.s* | 7 | 0.997 | Zhepi33 | cultivated | *H.v* | 4 | 0.995 |
| XZ121 | wild | *H.s* | 1 | 0.994 | Zheda9 | cultivated | *H.v* | 4 | 0.996 |
| XZ122 | wild | *H.a* | 6 | 0.923 | Zheda8 | cultivated | *H.v* | 4 | 0.982 |
| XZ123 | wild | *H.s* | 1 | 0.995 | B1052 | cultivated | *H.v* | 4 | 0.826 |
| XZ124 | wild | *H.s* | 1 | 0.992 | B1342 | cultivated | *H.v* | 2 | 0.592 |

Note: *H.s*, *H. vulgare* L. ssp. spontaneum; *H.a*, *H. vulgare* L. ssp. agriocrithum; *H.v, H. vulgare* L. ssp. vulgare; int, intermediate form with partial sterility for lateral spikelets.
